# Temporal changes in frequency of severe hypoglycemia treated by emergency medical services in types 1 and 2 diabetes: a population-based data-linkage cohort study

**DOI:** 10.1186/s40842-017-0045-0

**Published:** 2017-08-15

**Authors:** Huan Wang, Peter T. Donnan, Callum J. Leese, Edward Duncan, David Fitzpatrick, Brian M. Frier, Graham P. Leese

**Affiliations:** 1 Dundee Epidemiology and Biostatistics Unit, Population Health Sciences, University of Dundee, The Mackenzie Building, Kirsty Semple Way, Dundee, DD2 4BF UK; 2 University of Edinburgh, Faculty of Medicine, Edinburgh, UK; 3 NMAHP Research Unit, University of Stirling, Stirling, UK; 4 Scottish Ambulance Service, National Headquarters, Edinburgh, UK; 5 BHF Centre for Cardiovascular Science, The Queen’s Medical Research Institute, University of Edinburgh, Edinburgh, UK; 6 School of Medicine, Ninewells Hospital and Medical School, Dundee, UK

**Keywords:** Diabetes, Hypoglycemia, Emergency medical care, Insulin, Sulfonylurea

## Abstract

**Background:**

Almost 20 years ago, the frequencies of severe hypoglycemia requiring emergency medical treatment were reported in people with types 1 and 2 diabetes in the Tayside region of Scotland. With subsequent improvements in the treatment of diabetes, concurrent with changes in the provision of emergency medical care, a decline in the frequency of severe hypoglycemia could be anticipated. The present population-based data-linkage cohort study aimed to ascertain whether a temporal change has occurred in the incidence rates of hypoglycemia requiring emergency medical services in people with types 1 and 2 diabetes.

**Methods:**

The study population comprised all people with diabetes in Tayside, Scotland over the period 1 January 2011 to 31 December 2012. Patients’ data from different healthcare sources were linked anonymously to measure the incidence rates of hypoglycemia requiring emergency medical services that include treatment by ambulance staff and in hospital emergency departments, and necessitated hospital admission. These were compared with data recorded in 1997–1998 in the same region.

**Results:**

In January 2011 to December 2012, 2029 people in Tayside had type 1 diabetes and 21,734 had type 2 diabetes, compared to 977 and 7678, respectively, in June 1997 to May 1998. In people with type 2 diabetes, the proportion treated with sulfonylureas had declined from 36.8 to 22.4% (*p* < 0.001), while insulin-treatment had increased from 11.7 to 18.7% (*p* < 0.001). The incidence rate of hypoglycemia requiring emergency medical treatment had significantly fallen from 0.115 (95% CI: 0.094–0.136) to 0.082 (0.073–0.092) events per person per year in type 1 diabetes (*p* < 0.001), and from 0.118 (0.095–0.141) to 0.037 (0.003–0.041) in insulin-treated type 2 diabetes (*p* = 0.008). However, the absolute annual number of hypoglycemia events requiring emergency treatment was 1.4-fold higher.

**Conclusions:**

Although from 1998 to 2012 the incidences of hypoglycemia requiring emergency medical services appeared to have declined by a third in type 1 diabetes and by two thirds in insulin-treated type 2 diabetes, because the prevalence of diabetes was higher (2.7 fold), the number of severe hypoglycemia events requiring emergency medical treatment was greater.

## Background

Hypoglycemia remains a common side-effect of the treatment of diabetes with insulin despite the introduction of novel insulins, formulations and methods of delivery, coupled with advances in glycemic monitoring. The epidemiology of severe hypoglycemia (defined as any event requiring help for recovery) has been well documented in western countries [1], but is much more common in many parts of the world than had been recognised previously [2], as is non-severe (self-treated) hypoglycemia, particularly during sleep [1]. In unselected populations with type 1 diabetes in northern Europe the incidence of severe hypoglycemia has been reported to vary from 1.1 to 3.2 events per person per year [1, 3–8]. Population-based studies of emergency medical services utilisation [9] have reported the incidence of hypoglycemia requiring emergency treatment in people with type 1 diabetes to be between 0.08 and 0.49 events per person per year. Severe hypoglycemia events in people with diabetes account for 0.5% to 1.02% of all emergency ambulance call outs in the UK per annum [10–13] where the frequency of severe hypoglycemia and its treatment by emergency medical services has been surveyed by opportunistic examination of ambulance records [10, 11]. However, the accuracy and validity of many such surveys were limited by inadequate study design and retrospective review of incomplete data.

Almost 20 years ago, the frequencies of severe hypoglycemia events that required the assistance of emergency medical services from primary care, ambulance, hospital emergency and inpatient medical services were recorded prospectively in detail over a 12-month period (June 1997 to May 1998 inclusive) in people with types 1 and 2 diabetes in the region of Tayside in Scotland [12]. The incidences of severe hypoglycemia events requiring emergency medical treatment were 0.115 events per person per year in type 1 diabetes, 0.118 in insulin-treated type 2 diabetes and 0.009 in people with type 2 diabetes taking sulfonylureas, with respective prevalence of 7.1%, 7.3%, and 0.8%. Many more self-reported hypoglycemia events are treated by family members, friends or colleagues who do not request help from the emergency medical services [3, 12] and an incidence of 1.15 events per person per year in type 1 diabetes and 0.35 events per person per year in insulin-treated type 2 diabetes [12], were reported in the same region. Around 63% to 73% people who experienced severe hypoglycemia events remain at home following treatment by ambulance paramedics [10, 11, 13], which influences the frequency of treatment in hospital emergency departments (EDs) and hospital admission. With subsequent improvements in the treatment of diabetes, combined with changes in methods of providing emergency medical care, it could be anticipated that the frequency of severe hypoglycemia events requiring emergency treatment would have declined despite more intensive management of diabetes being applied to attain stricter glycemic control.

The present study aimed to re-measure the incidence rates of severe hypoglycemia in types 1 and 2 diabetes that had required emergency medical services in the same region of Tayside over the period 1 January 2011 to 31 December 2012, and by comparison with the incidence rates documented in 1997–1998, to ascertain whether these had changed significantly over time.

## Methods

### Data sources and linkage

Data were derived from several sources. The Scottish Care Information-Diabetes (SCI-Diabetes) provides an integrated shared electronic patient record to support the management of people with diabetes within the National Health Service (NHS) in Scotland. It includes information on type of diabetes, date of diagnosis and treatment modality. The Scottish Ambulance Service electronic patient records comprise the date of the event, the symptoms, diagnosis of hypoglycemia and treatment administered by ambulance clinicians to individual patients, which can identify emergency call-outs related to hypoglycemia. Data on admissions to EDs with hypoglycemia were obtained from Information Services Division Scotland. The Scottish Morbidity Register (SMR01) dataset collects episode level data on hospital inpatient and day case admissions from hospitals in Scotland. It provides information on dates of admission and discharge, and medical conditions, which can be used to identify hospital admission associated with hypoglycemia. Prescribing data were linked with diabetes drugs with British National Formulary (BNF) codes, to provide detailed information on the drugs dispensed. Data on social deprivation were available for the 2011–12 cohort using the Scottish index of multiple deprivation (SIMD) which is derived from the postcode via the community health index register [14]. The SIMD categories patients into five divisions of deprivation.

Data were collected and collated by the Health Informatics Centre in University of Dundee that conforms to ISO27001. Data was linked using the Community Health Index number that is used universally in the NHS with over 99% of accuracy for people with diabetes in Scotland.

### Study design and statistical analyses

The period of study was from 1 January 2011 to 31 December 2012, inclusive, and similar methodology was used to that employed in the previous study [12], enabling direct comparisons of incidence rates of severe hypoglycemia to be made with 1997–1998. The study population comprised all people with diabetes in Tayside, Scotland. All events of hypoglycemia that required emergency medical services (ambulance, ED attendance, and hospital admission) during the study period were identified. The incidence of hypoglycemia was analysed separately by type of diabetes. Type 1 diabetes was defined by requiring insulin within 6 months of diagnosis and not taking any oral glucose-lowering medication other than metformin. All other participants were considered to have type 2 diabetes and were further classified by the type of medications being prescribed in order of their propensity to induce hypoglycemia:

- Agents that do not usually cause hypoglycemia, (i.e. metformin, pioglitazone, dipeptidyl peptidase-4 (DPP-4) inhibitors, sodium-glucose co-transporter-2 (SGLT-2) inhibitors, glucagon-like peptide-1 (GLP-1) agonists).
- Oral medications that may cause hypoglycemia, i.e. sulfonylureas or glinides. This could be in combination with medication in category a).
- Treatment with insulin. This could be in combination with a medication in either category a) or b).

The incidence rates were expressed as events per person per year. The 95% confidence intervals were calculated based on the maximum likelihood method for Poisson-distributed observations. The incidence rate ratios (IRRs) along with their 95% confidence intervals and *p* values were calculated to assess the significance of differences between the two populations in the same region but at a different time period. All the analyses were conducted in R version 3.2.5.

## Results

### Study cohort

A substantial increase in the prevalence of diabetes had occurred in Tayside, Scotland during the 14 years separating the two surveys. In June 1997 to May 1998, of the 367,051 residents of Tayside, 8655 people had diabetes (crude overall prevalence = 2.4%), with 977 of 8655 (11.3%) having type 1 diabetes and 7678 (88.7%) having type 2 diabetes (Table 1). The present study examined data from 23,763 people with diabetes (mid-year population estimate in Tayside in 2012 = 411,749, crude overall prevalence of diabetes during the 2 years from 1 January 2011 to 31 December 2012 = 5.8%), which included 2029 with type 1 diabetes (8.5%) and 21,734 with type 2 diabetes (91.5%).

**Table 1:** People with diabetes and number of hypoglycemia events requiring emergency medical services in Tayside, Scotland

	Type 1 diabetes	Type 2 diabetes	Total
June 1997 – May 1998			
People with diabetes	977 (11.3%)	7678 (88.7%)	8655 (100%)
Mean age at study	33	66	--
Mean duration of diabetes (years)	17	8	--
People with severe hypoglycemia	69	91	160
Severe hypoglycemia events	112	132	244
1 January 2011–31 December 2012			
People with diabetes	2029 (8.5%)	21,734 (91.5%)	23,763 (100%)
Mean age at study	40	68	--
Mean duration of diabetes (years)	18	7	--
People with severe hypoglycemia	187	280	467
Severe hypoglycemia events	319	383	702

Between June 1997 and May 1998, 69 of 977 people with type 1 diabetes had experienced 112 hypoglycemia events requiring emergency medical services, while 91 of 7678 people with type 2 diabetes had been treated for 132 events. From 1 January 2011 to 31 December 2012, a total of 702 events of hypoglycemia requiring emergency medical services were recorded in 467 people with diabetes (Table 1). Some individuals had experienced recurrent episodes of hypoglycemia; 187 people with type 1 diabetes had experienced 319 events and 280 people with type 2 diabetes had experienced 383 events.

From June 1997 to May 1998, among the 7678 people with type 2 diabetes, 901 (11.7%) were being treated with insulin (Table 2), 2823 (36.8%) were being treated with sulfonylureas (group b), and 3954 (51.5%) were being treated with non-insulin secretagogues alone (group a). From 1 January 2011 to 31 December 2012, a significantly higher proportion of people with type 2 diabetes were receiving treatment with non-secretagogues (58.9%) or insulin (18.7%), but fewer were being treated with secretagogues (22.4%).

**Table 2:** Number of people (%) with type 2 diabetes and different types of treatment, including a) non-secretagogues e.g. metformin, pioglitazone, DPP-4 inhibitor, SGLT-2 inhibitor, GLP-1 agonist; b) secretagogues e.g. sulfonylureas and glinides; and insulin treated type 2 diabetes

Group of people with type 2 diabetes	Number of people (%)		Proportion difference (95% CI)	*P* value
	June 1997 – May 1998 (*n* = 7678)	1 Jan 2011–31 Dec 2012 (*n* = 21,734)		
a)	3954 (51.5%)	12,812 (58.9%)	0.075 (0.062–0.087)	< 0.001
b)	2823 (36.8%)	4859 (22.4%)	−0.144 (−0.156 – −0.132)	< 0.001
Insulin	901 (11.7%)	4063 (18.7%)	0.070 (0.061–0.078)	< 0.001

### Incidence of severe hypoglycemia

The overall annual incidence rates of severe hypoglycemia events requiring treatment by the emergency medical services during the 2 years of the survey are shown for the different treatment groups in Table 3. This was highest in people with type 1 diabetes (0.082 events per person per year, 95% CI: 0.073–0.092) and lowest in those with type 2 diabetes who were taking oral glucose-lowering medications that were not insulin secretagogues (group a). Among people with type 2 diabetes, the insulin-treated patients had a much higher incidence than those receiving other types of treatment. By comparison with the period of June 1997 to May 1998, significantly lower incidence were observed in people with type 1 diabetes (IRR = 0.720, 95% CI: 0.580–0.892, *p* < 0.001) and people with type 2 diabetes receiving insulin (IRR = 0.314, 95% CI: 0.249–0.395, *p* = 0.008). The IRRs for the groups receiving secretagogues (group a) and non-secretagogues (group b) were not calculated because the number of hypoglycemia events in these two groups were not currently available for the data from June 1997 to May 1998.

**Table 3:** Incidence rates of hypoglycemia requiring emergency medical services. (Rates for patients with type 1 diabetes, type 2 diabetes treated with a) non-secretagogues, b) secretagogues, or insulin)

Group	June 1997–May 1998	1 January 2011–31 December 2012			
	Incidence rate^a^ (95% CI)	Events (person-years)	Incidence rate^a^ (95% CI)	IRR^b^ (95% CI)	*P* value
Type 1	0.115 (0.094–0.136)	319 (3867.35)	0.082 (0.073–0.092)	0.720 (0.580–0.892)	< 0.001
Type 2 a)	0.001 (0.000–0.002)	50 (22,100.36)	0.002 (0.002–0.003)	--	--
b)	0.009 (0.006–0.013)	51 (9318.80)	0.005 (0.004–0.007)	--	--
Insulin	0.118 (0.095–0.141)	282 (7601.69)	0.037 (0.033–0.041)	0.314 (0.249–0.395)	0.008
All type 2	0.017 (0.014–0.020)	383 (39,020.86)	0.010 (0.009–0.011)	0.571 (0.468–0.696)	< 0.001

^a^Incidence rate is expressed as events per person per year. ^b^ *IRR* incidence rate ratio

No information was available on ethnicity but the prevalence of ethnic minorities is low at ≤3% in Tayside, Scotland, and has not changed in the time between these two surveys in what is a very stable population. Data on social deprivation were not available from 1997 to 1998, but data from 2011 to 2012 showed an increasing trend in the incidence rate of severe hypoglycemia in association with social deprivation in type 1 diabetes (Table 4), but a similar trend was not observed for different social deprivation categories in insulin-treated type 2 diabetes.

**Table 4:** Incidence rates of hypoglycemia requiring emergency medical services in people with type 1 and insulin-treated type 2 diabetes across different categories of Scottish index of multiple deprivation

Social deprivation	Incidence rates of severe hypoglycemia (95% CI)	
	Type 1 diabetes	Insulin-treated type 2 diabetes
1 (most deprived)	0.120 (0.093–0.147)	0.044 (0.033–0.054)
2	0.103 (0.078–0.128)	0.048 (0.036–0.059)
3	0.089 (0.069–0.108)	0.024 (0.016–0.031)
4	0.066 (0.051–0.081)	0.037 (0.029–0.045)
5 (least deprived)	0.041 (0.025–0.058)	0.032 (0.021–0.044)

### Emergency medical services utilized

**Fig. 1:**
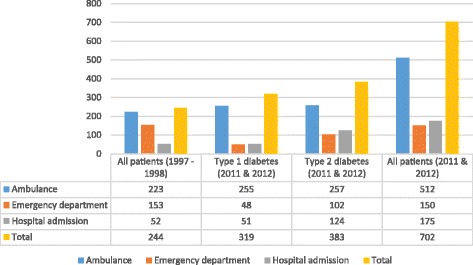
Number of hypoglycemia events requiring emergency medical services

The number of hypoglycemia events requiring different emergency medical services from 1 January 2011 to 31 December 2012 are shown in Fig. 1. Of the 702 events, 512 required emergency attendance by the ambulance service, 150 were treated in the hospital emergency department, and 175 events resulted in direct or indirect hospital admission. Any combination of these three emergency medical services was utilized in 116 hypoglycemia events. The proportion requiring inpatient hospital treatment had slightly increased from 21% (52 of 244 events) in 1997–1998 to 25% (175 of 702 events) in the period from 1 January 2011 to 31 December 2012, whereas usage of the ambulance service and of treatment in an emergency department was less frequent, decreasing from 91% (223 of 244) and 63% (153 of 244), respectively, to 73% (512 of 702) and 21% (150 of 702). Overall, 124 of 383 (32%) events in type 2 diabetes and 51 of 319 (16%) events in type 1 diabetes resulted in hospital admission. In general, a 1.4-fold increase in the total number of severe hypoglycemia events (244 events in 1997–1998 to an average of 351 events per year in 2011 and 2012) was recorded, compared to a 2.7-fold increase in the total diabetes population during the same period from 8655 to 23,763.

## Discussion

While severe hypoglycemia remains a major clinical problem, particularly for people treated with insulin, this may be declining in some patient groups. The present population-based, data-linkage study within a single region of Scotland has demonstrated that a lower incidence rate of severe hypoglycemia had required treatment by emergency medical services compared to the frequency observed 14 years earlier. While the incidence rate of severe hypoglycemia in people with type 1 diabetes had declined by about 30% (IRR = 0.720, 95% CI: 0.580–0.892, *p* < 0.001), a more prominent fall was observed in people with insulin-treated type 2 diabetes, which was almost 70% lower (IRR = 0.314, 95% CI: 0.249–0.395, *p* = 0.008). However, although the incidence of severe hypoglycemia had declined, the absolute number of events had increased by 1.4 fold, in association with a 2.7-fold rise in the size of the diabetes population in Tayside. A similar finding of a lower incidence of severe hypoglycemia but a higher absolute number requiring treatment in hospital was observed over a 10-year period in a study in England [15], while a higher absolute number of severe hypoglycemia events was also reported in a German study [16]. This indicates that the overall demand on the emergency medical services to treat severe hypoglycemia is rising, despite a decline in the incidence rate of severe hypoglycemia requiring emergency medical treatment.

The prevalence of diabetes in Tayside, Scotland in 1997–1998 was identified from an accurately recorded population-based data-linkage study [17]. While the greater number of people with diabetes in 2011 and 2012 compared to 1997–1998 probably reflects a genuine increase in prevalence of the disorder, it may partly result from better case ascertainment, with fewer people having undiagnosed diabetes and the observed prevalence is consistent with national prevalence statistics. The mean duration of type 2 diabetes has also declined from 1998 to 2011 and 2012, from 8 to 7 years, consistent with earlier diagnosis.

The lower incidence rate of severe hypoglycemia in type 1 diabetes that has now been observed may be a consequence of more intensive diabetes education in Tayside since 1998 [18] along with the greater use of glucose monitoring and insulin pumps, and the introduction of newer insulin analogues, which are associated with a lower rate of hypoglycemia [19], particularly at night. Although an overall reduction in the incidence rate of severe hypoglycemia may have been offset by greater efforts to improve glycemic control, the mean HbA1c in people with hypoglycemia in 2011–2012 were higher than in 1997–1998 for both type 1 and type 2 diabetes (Table 5). Poorer average glycemic control could be one explanation as to why the incidence rate of severe hypoglycemia events had declined, although in recent years no deterioration in glycemic control has been evidence in all patients with type 1 diabetes in Scotland [20]. The more likely explanation is that it may be easier to achieve good glycemic control without suffering hypoglycemia, and thus hypoglycemia is relatively more common in patients with erratic control and higher mean HbA1c. The assumption that an inverse linear relationship exists between the frequency of severe hypoglycemia and HbA1c concentration is not supported by cross-sectional studies of both type 1 [21] or type 2 diabetes [22], in which many people who experience recurrent severe hypoglycemia have elevated HbA1c concentrations. This is consistent with severe hypoglycemia being relatively common in people whose glycemic control is poor.

**Table 5:** Mean HbA1c levels with 95% confidence intervals in groups of people with types 1 or 2 diabetes who have had (or have not had) hypoglycemia events requiring emergency medical services. b) Secretagogues e.g. sulfonylureas and glinides

	Severe hypoglycemia	June 1997–May 1998		January 2011–December 2012	
		Number of people	Mean HbA1c (95% CI)	Number of people	Mean HbA1c (95% CI)
Type 1	No	908	7.93 (7.76–8.10)	1671	9.19 (5.45–12.93)
	Yes	69	7.77 (7.29–8.25)	182	9.00 (5.76–12.23)
Type 2 b)	No	2800	7.16 (7.08–7.23)	4772	7.93 (4.81–11.05)
	Yes	23	8.00 (7.10–8.91)	47	7.60 (4.72–10.48)
Insulin	No	835	8.23 (8.09–8.37)	3390	8.86 (5.16–12.56)
	Yes	66	7.87 (7.47–8.28)	183	8.49 (4.97–12.02)
All diabetes	No	8495	7.19 (7.14–7.24)	20,953	7.67 (4.21–11.13)
	Yes	160	7.85 (7.57–8.14)	454	8.47 (4.98–11.95)

In addition, the mean HbA1c values for 1997–98 and 2011–12 are not directly comparable as the ascertainment of HbA1c measurements were approximately 80% and 91% respectively, and patients not engaging with medical services are more likely to have poor glycemic control and are less likely to have HbA1c measured regularly. The explanation for the increasing age of patients with type 1 diabetes having hypoglycemia may be similar, with a greater proportion of younger patients receiving more modern insulins and or using continuous subcutaneous insulin infusion with an insulin pump.

In the 14 years between the two studies the proportion of people with type 2 diabetes treated with insulin had risen significantly from 11.7 to 18.7% (Table 2), and the absolute number of severe hypoglycemia events had increased from 132 to an average of 191 events per year. Despite the increase in the absolute number of events, the incidence rate of severe hypoglycemia in people with insulin-treated type 2 diabetes requiring emergency medical treatment was significantly lower and may have been promoted by changes in clinical management. These include more comprehensive patient education, the earlier use of insulin while significant β-cell function is still preserved, which is associated with a lower risk of hypoglycemia [23], and the earlier use of basal insulin in combination with oral agents, including DPP-4 inhibitors and GLP-1 agonists, which have a low risk of inducing hypoglycemia. The duration of insulin therapy is closely related to hypoglycemia risk in type 2 diabetes, which rises progressively as pancreatic β-cell function fails [3, 4, 24].

The incidence rate of severe hypoglycemia also declined in people with type 2 diabetes treated with sulfonylureas. This was associated with a decline in the use of sulfonylureas from 36.8 to 22.4%, and may reflect avoidance of their use in people at high risk of hypoglycemia. People with type 2 diabetes who experienced severe hypoglycemia were twice as likely to require hospital admission than those with type 1 diabetes (32% vs 16%), which has been reported by another British study [25]. People with type 2 diabetes are more likely to be older and more frail, both being risk factors that are associated with severe hypoglycemia [26, 27].

During the period of the present study, the ambulance service treated more episodes of severe hypoglycemia without the need to transfer patients to hospital. The increase in non-conveyance may have resulted from the introduction in non-conveyance clinical guidelines for post-hypoglycemia patients in 2005 [28].

The present study has some limitations. If an ambulance had been called after a self-treated episode of hypoglycemia had resolved, such an incident may not have been recorded as a severe hypoglycemia event. Hypoglycemia treated by General Practitioners (GP) would not have been recorded. Some cases may have been missed if the patients who had experienced severe hypoglycemia had presented with another principal complaint such as chest pain or the consequences of trauma, and blood glucose had not been measured.

The present study indicates that around 9% (187 of 2029) of people with type 1 diabetes experienced severe hypoglycemia that required emergency medical services, which compares to 13% in a recent large prospective study [29] that relied on self-reported data. When using self-reported data the incidence rate of severe hypoglycemia in type 2 diabetes has varied between 0.1 and 0.7 events per person per year [1, 3, 6, 10, 24, 30]. This compares to incidence rates of emergency medical treatment in the present study of 0.01 events per person per year for all people with type 2 diabetes and 0.037 events per person per year for those with insulin-treated type 2 diabetes (Table 3). In other studies, the reported rates were much lower at 0.017 events [31] and 0.003 events [32] per person per year, respectively.

## Conclusions

The present large population-based study over a 2-year period has used data from different sources, and describes routine experience of the involvement of emergency medical services in the treatment of severe hypoglycemia. It suggests that the incidence rates of severe hypoglycemia requiring emergency medical services are declining both in types 1 and 2 diabetes, although the absolute number of events requiring emergency treatment is rising. While the burden on the emergency medical services is greater, with less duplication of service utilization and a significant reduction of the number of people attending hospital for treatment, provision may be more efficient than in previous years.

## Abbreviations

DPP-4: Dipeptidyl peptidase-4
GLP-1: Glucagon-like peptide-1
NHS: National Health Service
SCI-Diabetes: Scottish Care Information-Diabetes
SGLT-2: Sodium-glucose co-transporter-2
SMR: Scottish Morbidity Register
